# Do Not Waste Alcohol

**Published:** 1890-01

**Authors:** 


Do not waste Alcohol.—The alcohol used in washing microscopical sections, and in many other operations, should not be thrown away, but placed in a bottle labled "old alcohol," and used in the alcohol lamp, for washing balsam off of slides, for hardening animal specimens, and for numerous other purposes which will suggest themselves.

